# LncRNA LUADT1 sponges miR-195 to prevent cardiac endothelial cell apoptosis in sepsis

**DOI:** 10.1186/s10020-020-00228-5

**Published:** 2020-11-23

**Authors:** Zhimin Zhang, Mingzhu Lv, Xiang Wang, Zheng Zhao, Daolong Jiang, Lihua Wang

**Affiliations:** 1 Department of Critical Care Medicine, Affliated Dongfeng Hospital, Hubei University of Medicine, Shiyan, 442008 Hubei People’s Republic of China; 2 Department of Clinical Laboratory, Affliated Dongfeng Hospital, Hubei University of Medicine, Shiyan, 442008 Hubei People’s Republic of China; 3 Department of Children’s Medical Center, Affliated Dongfeng Hospital, Hubei University of Medicine, Shiyan, 442008 Hubei People’s Republic of China

**Keywords:** Sepsis, LUADT1, miR-195, Cardiac endothelial cells, Pim-1, Apoptosis

## Abstract

**Background:**

The oncogenic role of the newly identified lncRNA LUADT1 has been revealed in lung adenocarcinoma. It was reported that LUADT1 plays a critical role in multiple human diseases. This study was carried out to investigate the role of LUADT1 in sepsis.

**Methods:**

Sixty patients with sepsis and sixty healthy volunteers were recruited for this study. Plasma samples were collected from all participants. Human primary coronary artery endothelial cells were also used in this study. The expression of Pim-1, miR-195 and LUADT1 were detected by RT-qPCR. The interaction between miR-195 and LUADT1 was determined by overexpression experiments and luciferase activity assay. Cell apoptosis was detected by flow cytometry. The expression of apoptosis-related protein was detected by Western blotting.

**Results:**

Bioinformatics analysis revealed the potential interaction between LUADT1 and miR-195, which was confirmed by dual luciferase reporter assay. LUADT1 was downregulated in patients with sepsis. Moreover, LPS treatment downregulated the expression of LUADT1 in primary cardiac endothelial cells. Overexpression of LUADT1 and miR-195 did not affect the expression of each other in primary cardiac endothelial cells. Interestingly, overexpression of LUADT1 was found to upregulate the expression of Pim-1, a target of miR-195. In addition, it was found that overexpression of LUADT1 and Pim-1 reduced the enhancement effects of miR-195 on LPS-induced cardiac endothelial cell apoptosis.

**Conclusion:**

In summary, LUADT1 may protect cardiac endothelial cells against apoptosis in sepsis by regulating the miR-195/Pim-1 axis.

## Introduction

Sepsis is a common and lethal clinical syndrome that occurs when severe systemic responses of body to infection, resulting in injury to its own tissues and organs (Cohen et al. 2015). Sepsis mostly affects elderly and patients complicated with other severe diseases, such as cancer (Gotts and Matthay 2016). The development of sepsis causes the dysfunction of multiple organs and induces critical illness characterized by severe immune dysfunction and catabolism (Gotts and Matthay 2016). Multiple therapeutic approaches have shown promising efficacy in improving the conditions of sepsis animal model, and some approaches are under clinical validation (Hotchkiss and Karl 2003; Riedemann et al. 2003; Cronin et al. 1995). However, antibiotics, which are correlated with the high prevalence of resistance, are still the most commonly used drugs in the clinical treatment of sepsis (Liu et al. (2017)).

The identification of genetic factors involved in the development and progression of sepsis provides novel insights into the development of targeted therapies (Giamarellos-Bourboulis and Opal 2016; Flores 2015; Poll et al. 2017). Besides protein-coding genes, the development and progression of sepsis is also accompanied with changes in the expression of non-coding RNAs (ncRNAs), such as miRNAs (Vasilescu et al. 2017). For instance, in mice sepsis model, miR-195 is upregulated, and inhibition of miR-195 can reduce the injuries of multiple organs (Zheng et al. 2015), suggesting that downregulation of miR-195 may be a potential therapeutic target for sepsis. LUADT1 is a newly identified oncogenic gene long non-coding RNA (lncRNA) in lung adenocarcinoma (Qiu et al. 2015). Our bioinformatics analysis revealed the potential interaction between LUADT1 and miR-195. This study was therefore carried out to investigate the potential interaction between LUADT1 and miR-195 in sepsis.

## Materials and methods

### Research subjects

This study passed the revised board of the Affiliated Dongfeng Hospital, Hubei University of Medicine (DFLS-2019-007). Research subjects of the present study included 60 patients with sepsis and 60 healthy controls who were enrolled in aforementioned hospital between January and June of 2019, and their basic characteristics were shown in Table 1. Inclusion criteria for sepsis patients: (1) Sepsis confirmed according to American College of Chest Physicians/Society of Critical Care Medicine criteria; (2) Sepsis patients with at least two of the followings when arrival ER and treated > 6 h, BT > 38 °C or < 36 °C, HR > 90/min, RR > 20/min or PaCO_2_ < 32 mmHg, WBC count > 12 × 10^9^/L or < 4 × 10^9^/L or immature form > 10%. Age and sex matched healthy volunteers were recruited as healthy controls, and people with the following situations were excluded: systematic inflammatory disease, solid cancer or hematological malignancies, severe kidney or hepatic dysfunction, undergoing steroid or immunosuppressive therapy. Written form of the informed consent was signed by both patients and healthy controls.

**Table 1:** Basic characteristics of sepsis patients and healthy controls

Characteristics	sepsis patients (n = 60)	Healthy controls (n = 60)	P value
Age (year)	53.12 ± 6.9	54.37 ± 6.7	0.32
Gender (n/%)			
Male	41 (68.3%)	41 (68.3%)	
Female	19 (31.7%)	19 (31.7%)	
BMI (Kg/m^2^)	24.30 ± 3.23	23.54 ± 2.91	0.18
Albumin (g/L)	25.761 (22.346–34.975)	NA	
WBC (× 10^9^/L)	13.017 (4.129–28.438)	NA	
APACHE II score	19.6 ± 5.1	NA	
Source of sepsis (n/%)			
Respiratory tract infection	21 (35.0%)	NA	
Urinary tract infection	16 (26.7%)	NA	
Abdominal infection	9 (15.0)	NA	
Pneumonia	6 (10.0%)	NA	
Wound infection	5 (8.3%)	NA	
Others	3 (5.0%)	NA	

### Plasma and cardiac endothelial cells

All patients and healthy controls were subjected to blood extraction (5 ml) under fasting conditions. Blood samples were transferred to EDTA tubes, followed by centrifugation at 1,200 g at room temperature for 10 min to prepare plasma samples. Human primary coronary artery endothelial cells (HCAECs, ATCC® PCS-100-020™) were purchased from ATCC. Cells were cultured in Endothelial Cell Growth Medium MV containing 10% FBS. Cells culture conditions were: 37 ºC, 5% CO_2_ and 95% humidity. Cells were harvested at 75–85% confluence to perform the following experiments.

### Transient transfections

PcDNA3.1 vector expressing the full length of LUADT1 and Pim-1 cDNAs were constructed by GeneCopoeia (Guangzhou, China). Negative control (NC) miRNA, miR-195 mimic, antagomir and siPim-1 were synthesized by Sangon (Shanghai, China). Lipofectamine 2000 (Thermo Fisher Scientific) was used to transfect 10 nM vector or 50 nM miRNA into 10^6^ cells. Control (C) cells were un-transfected cells, and NC cells were transfected with empty vector or NC miRNA. Cells were harvested at 24 h post-transfection.

### Dual luciferase reporter assay

PGL3 Luciferase Reporter Vector (Promega) was used to construct LUADT1 vector (full length cDNA). Co-transfection of LUADT1 vector and miRNA NC or miR-195 mimic was performed through aforementioned methods. Dual-luciferase reporter assay kit (Promega) was used to measure the relative luciferase activity. Firefly luciferase activity was normalized to renilla luciferase.

### RNA preparation and qPCR

Total RNAs were extracted from both tissue samples and in vitro cultured cells using Trizol reagent (Invitrogen). For LPS treatment, HCAECs were cultured in cell culture medium containing 0, 100,150, 200 and 500 ng/ml LPS for 24 h before use. DNA eraser (Takara) was used to digest all RNA samples to remove genomic DNA. Reverse transcriptions (RTs) were performed using PrimeScript RT-polymerase (Takara) with total RNA samples as template. With cDNA samples as template, qPCR reaction mixtures were prepared using QuantiTect SYBR Green PCR Kit (QIAGEN). The expression levels of LUADT1 and Pim-1 mRNA were measured with GAPDH as endogenous control. Extraction of miRNAs from aforementioned samples and cells was performed using miRNeasy Mini Kit (QIAGEN). The expression levels of mature miR-195 were measured using All-in-One™ miRNA qRT-PCR Detection Kit (Genecopoeia) with U6 as endogenous control. All PCR reactions were performed in triplicate manner and fold changes of gene expression levels were calculated using 2^−ΔΔCt^ method.

### Western blot

Cells were collected at 24 h post-transfection and washed twice in PBS. Cells were then lysed in RIPA solution containing protease inhibitor (Sigma-Aldrich) to prepare cell lysates. BCA assay kit (Sigma-Aldrich) was used to measure protein concentrations. Protein samples were denatured in boiling water for 10 min. After that, equivalent amount of protein samples were separated by 10% SDS-PAGE gel, followed by gel transfer to PVDF membranes. Membranes were subsequently blocked in PBS containing 5% non-fat milk at room temperature for 2 h. Then membranes were incubated with primary antibodies of rabbit anti-GAPDH (ab9845, Abcam) and anti-Pim-1 (ab98004, Abcam), caspase3 (ab13847, Abcam) at 4 ºC for 12 h. After that, membranes were further incubated with HRP Goat Anti-Rabbit (IgG) secondary antibody (ab6721, Abcam) at 25 ºC for 2 h. ECL chemiluminescence kit (Pierce) was used to produce signals, which were normalized to GAPDH endogenous control using Image J v1.48 software.

### Flow cytometric analysis

Cells were harvested at 24 h post-transfection and cell cultures were performed using 6-cm tissue culture dishes with cell culture medium supplemented 150 ng/ml LPS for another 24 h. Pre-cold PBS was used to resuspend adherent cells. Alexa Fluor® 647/7-AAD apoptosis kit (BioLegend) was used to process cells with all steps performed following the manufacturer’s instructions. Flow cytometry (Becton–Dickinson) was performed to detect apoptotic cells.

### Statistical analysis

Data from 3 independent biological replicates were used to calculate the mean values, which were subjected to statistical analysis using SPSS software (version 18.0). Unpaired t test was used to explore the differences of two groups. Differences among multiple groups were analyzed by ANOVA (one-way) and Tukey test. *P* < 0.05 was considered as statistically significant. Correlations were performed using Pearson’s correlation coefficient.

## Results

### LUADT1 was downregulated in patients with sepsis

**Fig. 1:**
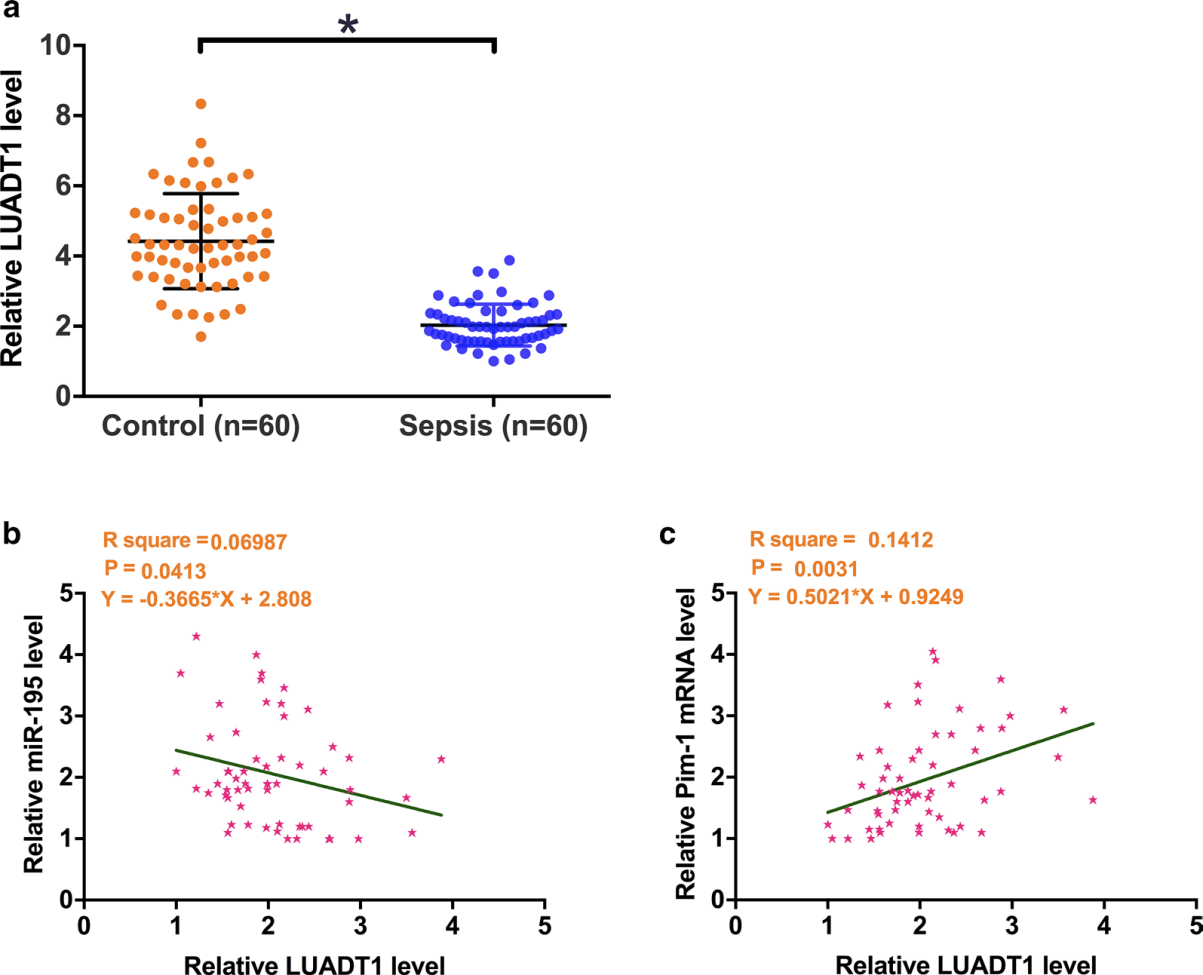
LUADT1 was downregulated in patients with sepsis. The expression of LUADT1 was analyzed by measuring the levels of LUADT1 in plasma from both sepsis patients (n = 60) and healthy controls (n = 60). Data were compared between two groups by unpaired t test. PCR reactions were repeated 3 times and mean values were presented (**a**). The mRNA expression levels of miR-195 and Pim-1 from sepsis patients were also measured by performing RT-qPCR. Correlations between LUADT1 and miR-195 (**b**)/Pim-1 mRNA (**c**) were analyzed by Pearson’s correlation coefficient. Mean values were used in analyses. *, *p* < 0.05

The expression of LUADT1 was detected in plasma from both sepsis patients (n = 60) and healthy controls (n = 60). It was observed that the expression levels of LUADT1 were significantly lower in sepsis patients then that in the control group (Fig. 1a, p < 0.05). Correlations between LUADT1 and miR-195 were analyzed by Pearson’ correlation coefficient. The results showed that the expression levels of LUADT1 were significantly and inversely correlated with the expression levels of miR-195 in sepsis patients (Fig. 1b, R^2^ = 0.0699, *p* < 0.05), while the expression levels of Pim-1 were positively correlated with that of LUADT1 (Fig. 1c, R^2^ = 0.1412, *p* < 0.05).

### LUADT1 and miR-195 can directly interact with each other

**Fig. 2:**
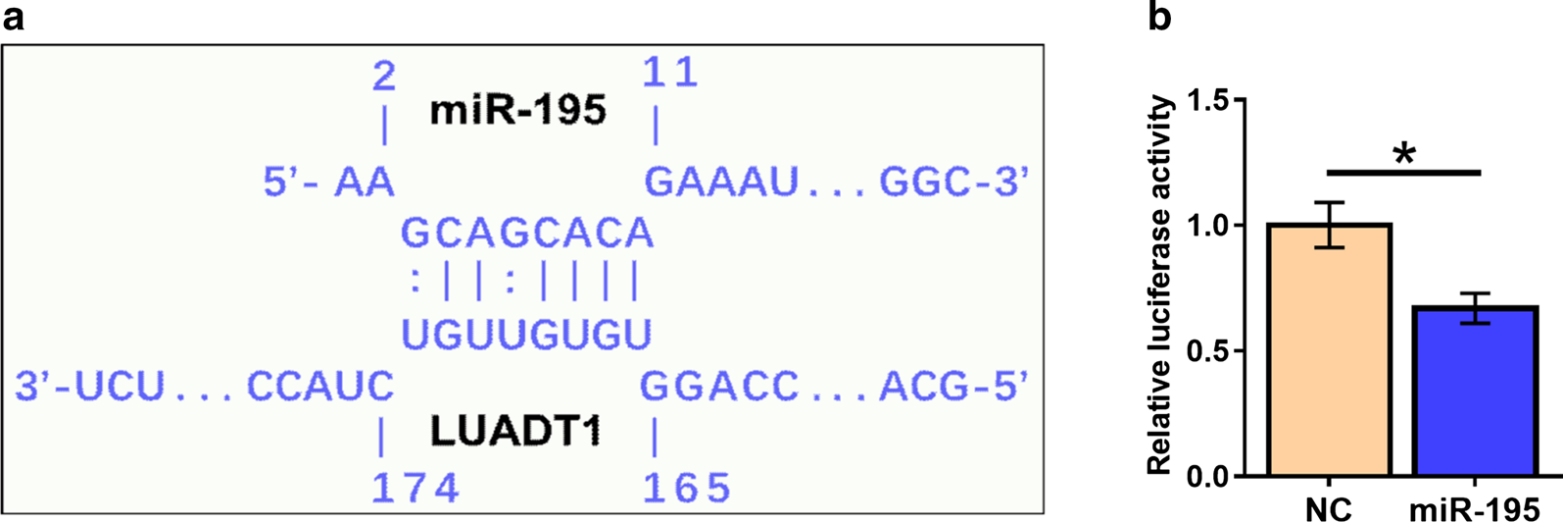
LUADT1 and miR-195 can directly interact with each other. The potential interaction between LUADT1 and miR-195 was analyzed by IntaRNA (https://rna.informatik.uni-freiburg.de/IntaRNA/Input.jsp) (**a**). Dual luciferase assay was performed by co-transfecting LUADT1 vector and miRNA NC (NC group) or LUADT1 vector and miR-195 mimic (miR-195 group) into HCAECs. Experiments were repeated 3 times and mean values were presented (**b**). *, *p* < 0.05

The potential interaction between LUADT1 and miR-195 was analyzed by the online program IntaRNA (https://rna.informatik.uni-freiburg.de/IntaRNA/Input.jsp) for RNA-RNA interaction. It was observed that LUADT1 and miR-195 could form strong base paring between each other (Fig. 2a). Dual luciferase reporter assay was performed to further analyze the interaction between them. Compared with HCAECs co-transfected with LUADT1 vector and miRNA NC (NC group), HCAECs co-transfected with LUADT1 vector and miR-195 mimic (miR-195 group) showed significantly lower relative luciferase activity (Fig. 2b, p < 0.05).

### Overexpression of LUADT1 and miR-195 did not affect the expression of each other

**Fig. 3:**
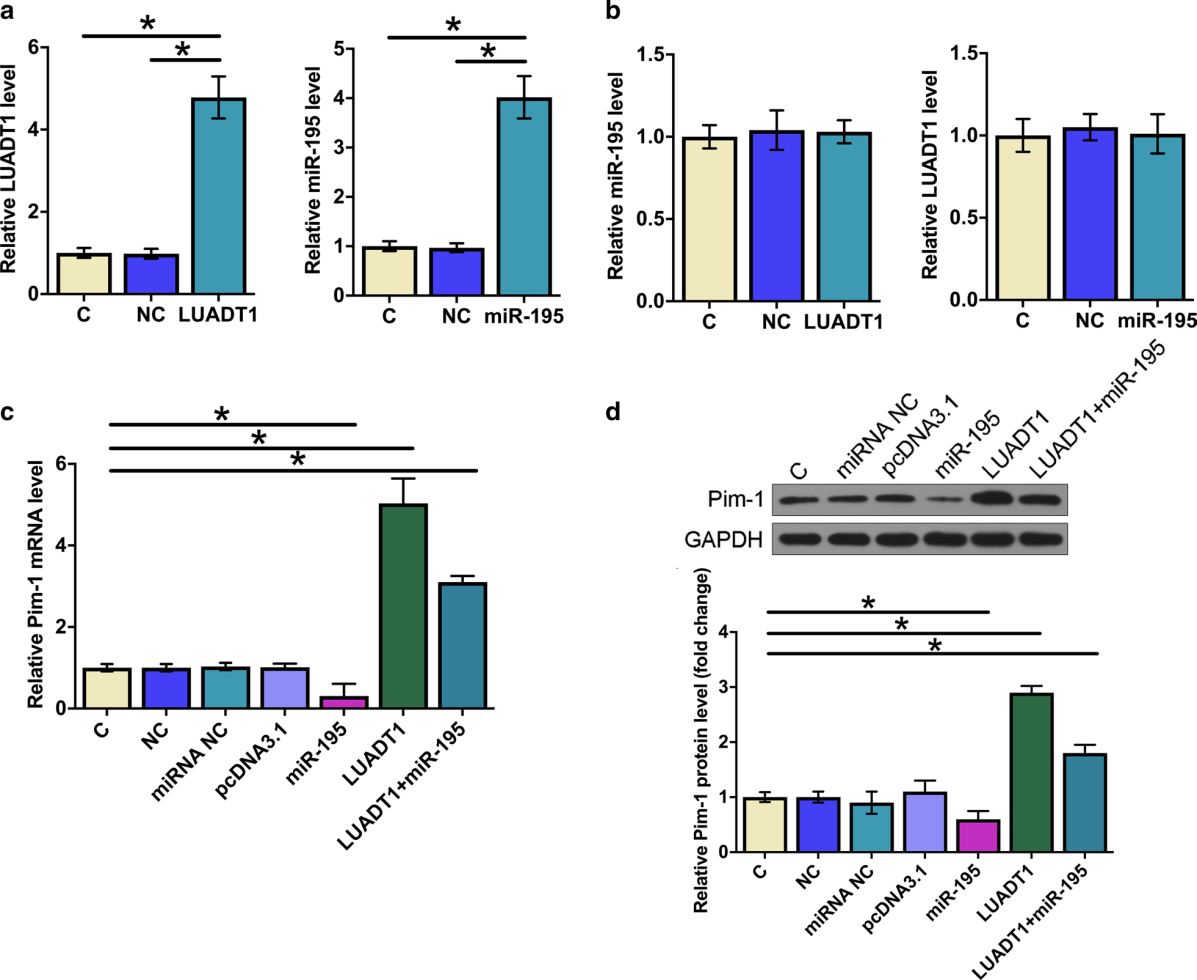
Overexpression of LUADT1 and miR-195 did not affect the expression of each other. HCAECs were transfected with LUADT1 expression vector and miR-195 mimic. LUADT1 and miR-195 overexpression was confirmed by RT-qPCR at 24 h post-transfection (**a**). The effects of LUADT1 and miR-195 overexpression on the expression of each other were also analyzed by RT-qPCR at 24 h post-transfection (**b**). The effects of overexpression of LUADT1 and miR-195 mimic on the expression of Pim-1, a downstream target of miR-195, at both mRNA and protein levels were analyzed by RT-qPCR (**c**) and western blot (**d**). Experiments were repeated 3 times, and data were expressed a mean value. *, *p* < 0.05

To further analyze the interaction between LUADT1 and miR-195, HCAECs were transfected with LUADT1 expression vector and miR-195 mimic. Overexpression of LUADT1 and miR-195 were confirmed by RT-qPCR at 24 h post-transfection (Fig. 3a, p < 0.05). Compared with the two control groups C and NC, overexpression of LUADT1 and miR-195 did not significantly alter the expression of each other (Fig. 3b, p < 0.05). RT-qPCR and Western blotting were performed to elucidate the effects of overexpression of LUADT1 and miR-195 on the expression of Pim-1, a downstream target of miR-195, at both mRNA and protein levels. Compared with C (un-transfected cells) and NC (empty pcDNA3.1 vector or NC miRNA transfection) groups, overexpression of LUADT1 upregulated, while overexpression of miR-195 downregulated Pim-1 expression at both mRNA (Fig. 3c, p < 0.05) and protein (Fig. 3d, p < 0.05) levels. Moreover, overexpression of miR-195 attenuated the effects of LUADT1 overexpression (*p* < 0.05).

### LUADT1 suppressed LPS-induced apoptosis of HCAECs through miR-195/Pim-1

**Fig. 4:**
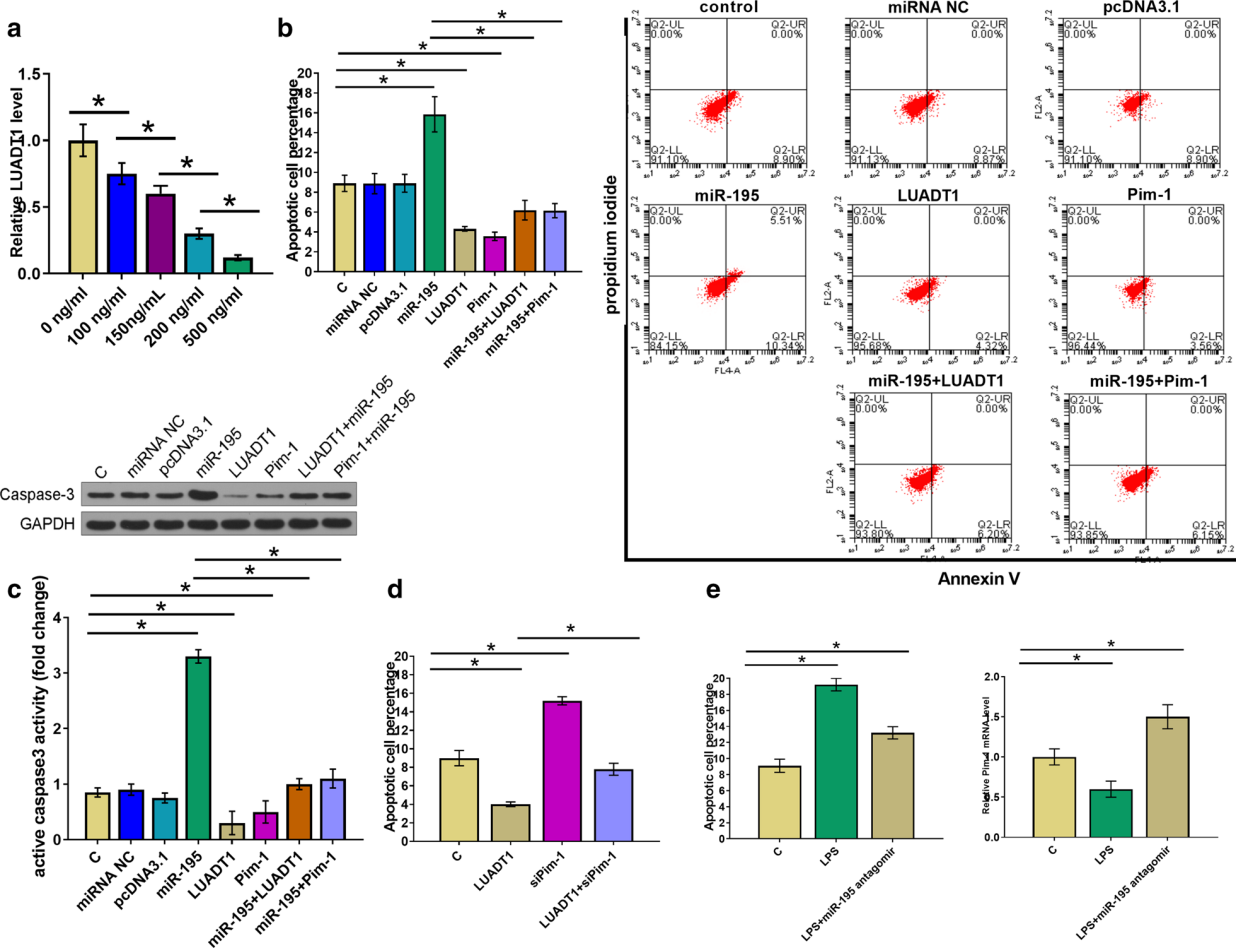
LUADT1 suppressed LPS-induced apoptosis of HCAECs through miR-195/Pim-1. HCAECs were cultured in cell culture medium containing 0, 100, 150, 200 and 500 ng/ml LPS for 24 h, followed by the measurement of the expression of LUADT1 by RT-qPCR (**a**). Cell apoptosis assay was performed to analyze the effects of LUADT1, miR-195 and Pim-1 overexpression on the apoptosis of LUADT1 treated by 150 ng/ml LPS (**b**). Caspase3 activity assay was detected by Western blotting (**c**). Apoptosis was detected after the overexpression of Pim-ko and LUADT1 (**d**). The expression of Pim-1 and apoptosis were examined after using miR-195 antagomir at 150 ng/mL LPS (**e**). All experiments were repeated 3 times and data were expressed as mean values. *, *p* < 0.05

HCAECs were cultured in cell culture medium containing 0, 100, 150, 200 and 500 ng/ml LPS for 24 h, followed by measuring the expression levels of LUADT1 by qPCR. It was observed that LPS treatment downregulated the expression of LUADT1 in a dose-dependent manner (Fig. 4a, p < 0.05). Cell apoptosis assay was performed to analyze the effects of LUADT1, miR-195 and Pim-1 overexpression on the apoptosis of HCAECs treated by 150 ng/ml LPS. Compared with C group and NC groups, cell apoptosis analysis showed that overexpression of LUADT1 and Pim-1 decreased, while overexpression of miR-195 increased the apoptotic rate of HCAECs. In addition, overexpression of miR-195 attenuated the effects of LUADT1 or Pim-1 overexpression (Fig. 4b, p < 0.05). Caspase 3 activity was consistent with the apoptosis results (Fig. 4c). Cell apoptosis was detected after the overexpression of Pim-ko and LUADT1, knock-down of Pim-1 decreased the HCAEC apoptosis (Fig. 4d). The expression of Pim-1 and the cell apoptosis were examined after adding miR-195 antagomir at 150 ng/mL LPS. It showed that the expression levels of Pim-1 were decreased after LPS treatment, while increased after miR-195 antagomir treatment (Fig. 4e).

## Discussion

**Fig. 5:**
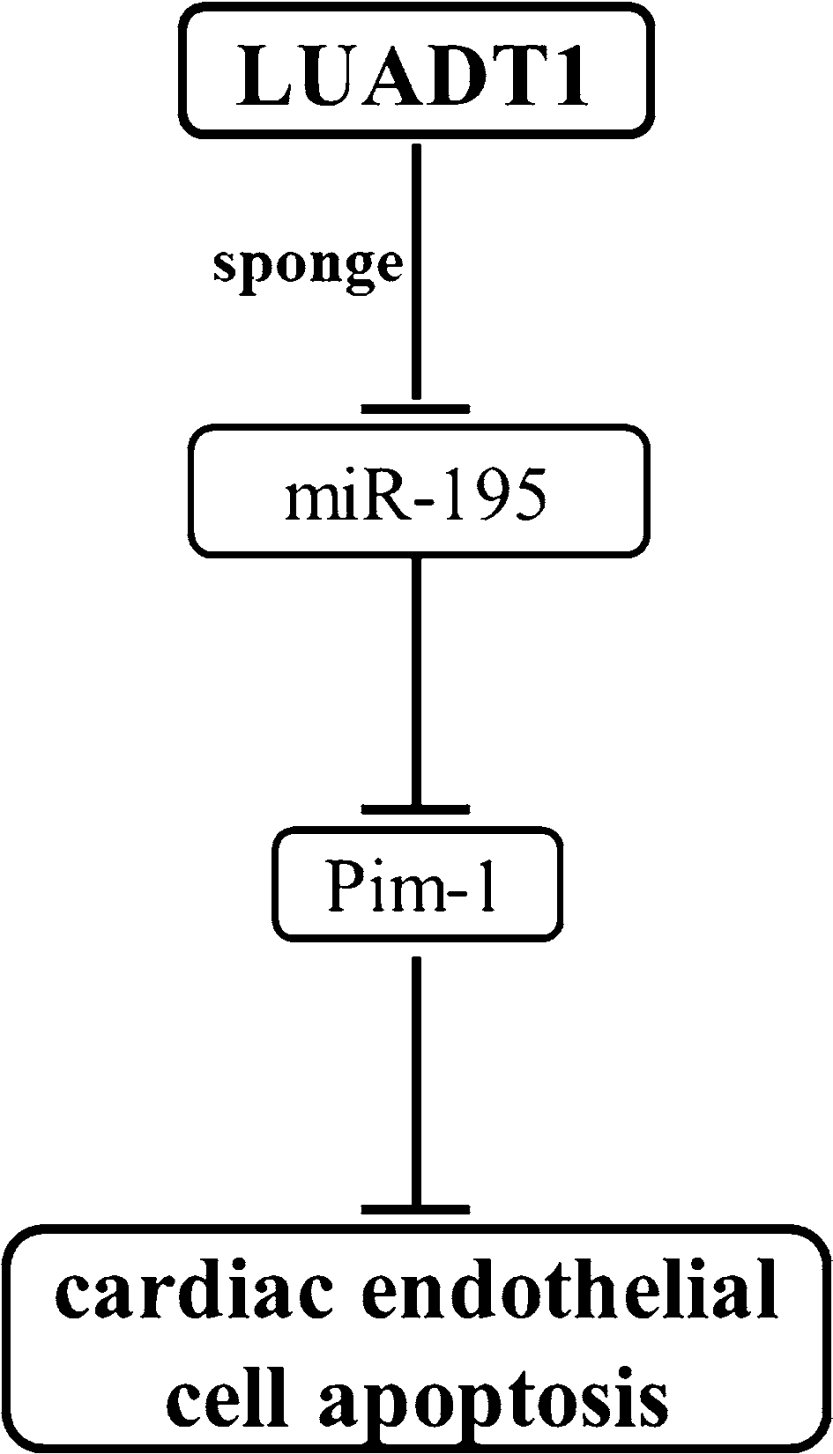
Schematic figure outlining the mechanism by which LUADT1 protects cardiac endothelial cells against LPS-induced apoptosis

The function of LUADT1 has only been investigated in lung adenocarcinoma (Qiu et al. 2015). It was observed that LUADT1 downregulated P27 by epigenetic pathway, thereby upregulating and promoting the proliferation of lung adenocarcinoma cells (Qiu et al. 2015). Our study revealed a new role of LUADT1 in sepsis. We found that LUADT1 was downregulated in sepsis and might regulate the miR-195/Pim-1 axis to inhibit LPS-induced of HCAEC apoptosis. This observation is of great significance for the clinical treatment of patients with sepsis (Fig. 5).

Heart is one of the most commonly affected organs by sepsis, and sepsis-induced heart failure leads to unacceptable high mortality rate. Sepsis-induced cardiomyopathy (SIC) is an infection-related cardiovascular disease. Studies have shown that at least 50% of patients with septic shock are clinically diagnosed with septic cardiomyopathy (Condor et al. 2017; Hong et al. 2018). Therefore, properly handling with the sepsis-induced heart failure became the urgent goal for all physicians worldwide. Previous studies have revealed that the apoptosis of cardiomyocyte and cardiac endothelial cells is involved in the pathological mechanism of cardiomyopathy (Uchida et al. 2010; Steiner and Lang 2017). In our study, overexpression of LUADT1 decreased the apoptotic rate of HCAECs under the treatment of LPS. Thus, LUADT1 may serve as a potential therapeutic drug for protecting patients’ hearts through the treatment of sepsis. However, animal model and clinical studies are needed to further verify the clinical applications of LUADT1. In addition, the protective effects of LUADT1 on other organs remain to be further elucidated.

The development and progression of sepsis is accompanied by changes in the expression of multiple miRNAs (Vasilescu et al. 2017). Some of the differentially expressed miRNAs have been reported to play critical roles in sepsis (Ge et al. 2018; Wu et al. 2018). For instance, overexpression of miR-214 improved cardiac function, reduced inflammation and myocardial injury (Ge et al. 2018). In contrast, overexpression of miR-223 is significantly correlated with inflammatory responses and poor prognosis of sepsis patients (Wu et al. 2018). In a recent study, miR-195 was overexpressed in mouse sepsis model, and downregulation of miR-195 inhibited injuries and cell apoptosis in multiple organs (Zheng et al. 2015). Consistently, our study further confirmed the role of miR-195 in promoting the apoptosis of HCAECs under the treatment LPS. Herein, miR-195 and LUADT1 were predicted to interact with each other. Although miR-195 and LUADT1 did not affect the expression of each other, overexpression of LUADT1 led to the upregulation of Pim-1, a target of miR-195. Pim-1 was reported to regulate cardiomyocyte survival and promote the proliferation of cardiac progenitor cells, which provides a perspective in combining LUADT1 and Pim-1 in heart protection (Muraski et al. 2007; Cottage et al. 2012). Our data supported the idea that LUADT1 might sponge miR-195 to upregulate Pim-1.

## Conclusions

In conclusion, LUADT1 is downregulated in sepsis and may regulate the miR-195/Pim-1 axis to inhibit the apoptosis of HCAECs induced by LPS.

## Data Availability

The analyzed data sets generated during the study are available from the corresponding author on reasonable request.

## Abbreviation

ncRNAs: Non-coding RNAs
